# Role of Pharmacology in Dentistry: A Review of Analgesics, Antibiotics, and Local Anesthetics

**DOI:** 10.7759/cureus.84889

**Published:** 2025-05-27

**Authors:** Anupam Datta, Bikramaditya Mukherjee, Shilpa Khullar Sood, Surupa Dutta, Rucha Barve, Unni Pympallil, Selvan Ravindran

**Affiliations:** 1 Department of Forensic Medicine and Toxicology, Agartala Government Medical College, Agartala, IND; 2 Department of Biochemistry, Manipal TATA Medical College, Jamshedpur, Jamshedpur, IND; 3 Department of Dentistry, Amrita Vishwa Vidyapeetham, Faridabad, Faridabad, IND; 4 Department of Periodontology, Buddha Institute of Dental Sciences and Hospital, Patna, IND; 5 Department of Endodontics, Dr. Barve's Dental Clinic, Pune, IND; 6 Department of Prosthodontics, Mahe Institute of Dental Sciences and Hospital, Mahe, IND; 7 Department of Medical and Health Sciences, Symbiosis International (Deemed University), Pune, IND

**Keywords:** analgesics, antibiotics, antimicrobial resistance, dental pharmacology, local anesthetics, pain management

## Abstract

This review explores the essential role of pharmacology in dentistry, emphasizing the use of analgesics, antibiotics, and local anesthetics in managing pain, controlling infections, and ensuring procedural comfort. The integration of these pharmacological agents has significantly advanced dental care, allowing for more effective and patient-friendly treatments. Nonsteroidal anti-inflammatory drugs (NSAIDs) and acetaminophen serve as the primary analgesics, while opioids, despite their efficacy in severe cases, require careful prescribing due to their high risk of dependency and adverse effects. Antibiotics are indispensable for treating odontogenic infections and preventing systemic complications, yet their overuse has contributed to the global challenge of antimicrobial resistance, necessitating stringent antibiotic stewardship in dental practice. Local anesthetics, particularly lidocaine and articaine, have revolutionized pain-free dentistry, with advancements such as liposomal formulations, buffered anesthetics, and computer-assisted anesthesia enhancing their effectiveness and safety. Despite these innovations, challenges such as opioid dependency, antibiotic resistance, and individual variability in anesthetic response underscore the need for personalized pharmacotherapy. Emerging research in pharmacogenomics and novel drug delivery systems holds promise for optimizing treatment outcomes by tailoring pharmacological interventions to individual patient profiles. Future directions should focus on integrating precision medicine, improving antimicrobial strategies, and developing safer, more efficient anesthetic techniques. By incorporating evidence-based prescribing practices and adopting innovative pharmacological approaches, dentistry can continue to evolve toward safer, more effective, and patient-centered care.

## Introduction and background

Modern dentistry depends heavily on pharmacology because this discipline enables pain management and infection control, along with patient comfort for dental treatment procedures. The integration of pharmacological agents into dental practice has produced a major transformation in patient care as clinicians now carry out standard and intricate procedures with minimized discomfort and reduced risk [1]. Dental pharmacology depends on three fundamental drug categories, which include local anesthetics, analgesics, and antibiotics that operate as separate entities to improve treatment effectiveness and patient recovery results. Medical drugs enable pain control by managing infections while boosting the clinical effectiveness of dental procedures that include extractions, root canal treatments, periodontal surgeries, and implant placements [2]. The appropriate utilization of drugs protects patients from dangerous systemic oral infection complications, including bacterial endocarditis, osteomyelitis, and other severe conditions [3]. The dental practice needs to adopt evidence-based prescribing approaches to tackle antibiotic resistance, together with opioid dependency and adverse drug effects [4].

Analgesics serve as the vital foundation for dental pain management since they treat both sudden and continuous pain that emerges from dental conditions and operative procedures. Medical professionals consider nonsteroidal anti-inflammatory drugs (NSAIDs) and acetaminophen as preferred treatments for dental pain since these medications effectively minimize pain and inflammation [5]. Healthcare providers administer opioid painkillers as a second option, but they do so with great caution due to dependency risks and adverse side effects. The treatment and prevention of odontogenic infections require antibiotics, particularly for patients with abscesses and pericoronitis as well as post-surgical complications [6]. The preventive use of antibiotics occurs for high-risk patients who possess prosthetic heart valves or immunosuppressive conditions to stop the development of serious infections such as infective endocarditis [7]. The excessive use of antibiotics by dental professionals has been a main factor in antimicrobial resistance development, so dental practitioners must follow antibiotic stewardship principles [8].

Local anesthetics serve an equally vital purpose because they provide painless dental procedures that improve patient compliance while reducing procedural anxiety. New-era dental pain management stems from two primary breakthroughs, which are lidocaine and articaine as amide-based anesthetics alongside innovative buffered agents and computer-driven anesthetic delivery methods [9].

The research evaluates pharmacology's function in dentistry through an assessment of analgesic, antibiotic, and local anesthetic clinical usages with their effectiveness combined with safety considerations. The assessment examines the operative mechanisms of these drugs while reviewing their prescription protocols and details the connected dangers involving antibiotic resistance patterns and opioid misuse incidents. The analysis focuses on modern developments within dental pharmacology as well as future trends to enhance evidence-based medication prescriptions, which ultimately benefits patient care quality.

## Review

Types of analgesics used in dentistry

The first-choice pain treatment in dentistry depends on non-opioid medications such as NSAIDs and acetaminophen, which present both effectiveness and safety properties. Ibuprofen, belonging to the NSAID family, blocks cyclooxygenase (COX) enzymes to decrease prostaglandin production, which helps reduce pain from inflammation, thus making it suitable for dental surgical pain and periodontal pain treatment [10]. These medications provide restricted pain relief to patients who do not have renal, cardiovascular, or gastrointestinal risks [11]. Acetaminophen functions as an effective painkiller for patients who cannot tolerate NSAIDs, although its high dose consumption raises hepatotoxicity risks [12].

**Figure 1:**
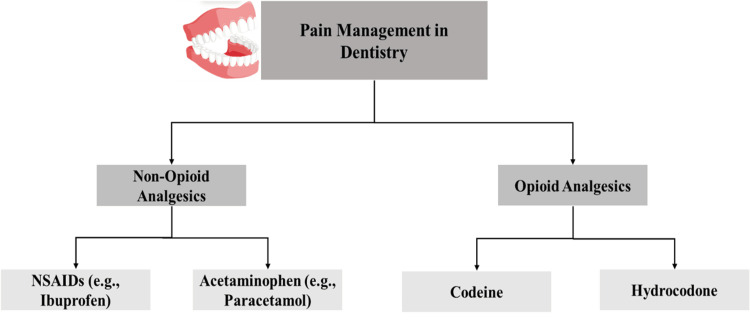
Types of Analgesics Used in Dentistry NSAIDs: nonsteroidal anti-inflammatory drugs Credit: The image was created by the authors.

Codeine and hydrocodone opioid analgesics should be prescribed only when patients experience severe dental pain that does not respond to NSAIDs or acetaminophen. The compounds activate opioid receptors throughout the central nervous system to control pain, yet pose major dangers such as addiction, together with respiratory failure and mental sedation [13]. The medical field utilizes opioid medications only in selected short-term applications. The use of NSAIDs or acetaminophen alongside opioids delivers improved pain reduction and decreases the amount of opioid medication as well as adverse effects [14]. The analgesics used in dentistry are illustrated in Figure 1.

The medical community supports the combination of NSAIDs with acetaminophen treatments over opioids for treating dental pain at moderate-to-severe levels. This treatment approach shows better results and safety characteristics, which reduces the need for opioid medications. The optimal method for dental pain management involves combining NSAIDs with acetaminophen, which corresponds to present opioid-sparing recommendations [1].

Mechanism of action and pharmacokinetics

The pain relief mechanisms of dental analgesics consist of different approaches between NSAIDs, acetaminophen, and opioids. The pain-relieving effects of NSAIDs occur because these drugs block cyclooxygenase (COX-1 and COX-2) enzymes, which leads to a reduction of prostaglandin synthesis and a subsequent decrease in inflammation, swelling, and pain sensitivity [15]. The main pain-relieving effect of acetaminophen occurs through its action on COX-3 enzymes in the CNS while showing minimal anti-inflammatory properties. The brain and spinal cord opioid receptors µ, κ, and δ enable opioids to block pain signals by diminishing substance P and glutamate, and other neurotransmitters [16].

**Figure 2:**
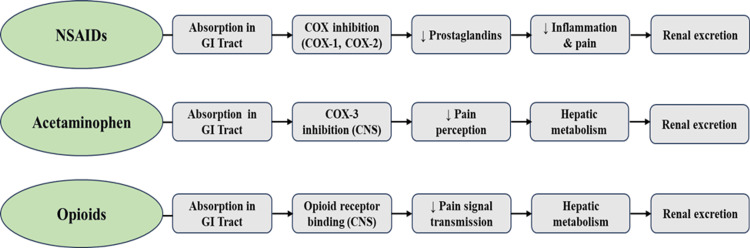
Mechanism of Action of Analgesics in Dentistry NSAIDs: nonsteroidal anti-inflammatory drugs; GI: gastrointestinal; CNS: central nervous system; COX: cyclooxygenase Credit: The image was created by the authors.

The pharmacokinetic profile shows that NSAIDs absorb quickly from the gastrointestinal tract, after which they become highly protein-bound before the liver processes them through the CYP2C9 enzyme while eliminating them through the kidneys [17]. Acetaminophen shows good absorption in the body, while the liver metabolizes it through glucuronidation and sulfation before renal excretion takes place. The liver (CYP3A4, CYP2D6) metabolizes opioids during the first-pass process while these drugs penetrate the CNS due to their lipophilic nature and experience renal elimination [18]. The mechanism of action of these three analgesics is illustrated in Figure 2.

Potential side effects and drug interactions

The clinical use of dentistry-related analgesics and local anesthetics is affected by multiple possible side effects and drug reactions. Long-term use of NSAIDs, including ibuprofen and naproxen, brings the risk of gastric ulcers as well as renal toxicity and cardiovascular risks [19]. Acetaminophen provides general safety benefits, but its hepatotoxicity risk increases when taken in excessive amounts or by people who consume alcohol chronically [20]. The opioid medications codeine and oxycodone cause respiratory problems alongside sedation effects, addiction risks, and require careful doctor supervision for prescription purposes [21]. When used in high doses, local anesthetics lidocaine and bupivacaine can result in systemic toxicity, prolonged numbness, and cardiotoxic effects according to the literature [22]. A summary of drug-related risks, drug-to-drug interaction contraindications, and clinical considerations is mentioned in Table 1 for dental practice analgesics and local anesthetics.

**Table 1:** Potential Side Effects and Drug Interactions of Commonly Used Analgesics and Local Anesthetics NSAIDs: nonsteroidal anti-inflammatory drugs; GI: gastrointestinal; CNS: central nervous system; ACE: angiotensin-converting enzyme; SSRIs: selective serotonin reuptake inhibitors; MAO: monoamine oxidase; ECG: electrocardiogram

Drug Class	Common Drugs	Potential Side Effects	Drug Interactions	Contraindications	Clinical Considerations
NSAIDs	Ibuprofen, naproxen	Gastric ulcers, GI bleeding	Anticoagulants (increase bleeding risk), corticosteroids (increase GI damage)	Peptic ulcer disease, kidney disease	Use gastroprotective agents if high GI risk
	Aspirin, celecoxib	Cardiovascular risks, renal toxicity	ACE inhibitors (decrease antihypertensive effect), lithium (increases toxicity)	Cardiovascular disease, aspirin allergy	Monitor renal and cardiovascular function
Acetaminophen	Paracetamol	Hepatotoxicity, nausea	Alcohol (increases liver toxicity), warfarin (increases bleeding risk)	Liver disease, chronic alcoholism	Limit the daily dose to avoid liver toxicity
		Hypersensitivity reactions	Barbiturates (increase metabolism), antiepileptics (increase toxicity)	Severe hepatic impairment, pregnancy	Avoid in patients with epilepsy or hypersensitivity
Opioids	Codeine, hydrocodone	Respiratory depression, constipation	Benzodiazepines (increase sedation), MAO inhibitors (increase respiratory depression)	Respiratory disorders, substance abuse	Use the lowest effective dose for the shortest duration
	Oxycodone, morphine	Sedation, addiction potential	SSRIs (increase serotonin syndrome risk), CNS depressants (increase sedation)	Head trauma, elderly patients	Monitor for signs of dependency
Local Anesthetics	Lidocaine	Allergic reactions, systemic toxicity	Beta-blockers (decrease efficacy), cholinesterase inhibitors (increase toxicity)	Allergy to amides, severe hepatic disease	Avoid in patients with heart conditions
	Articaine	Paresthesia, prolonged numbness	Epinephrine (increases cardiovascular effects), sulfonamides (increase toxicity)	Neurological disorders, pediatric use	Use cautiously in patients with nerve damage
	Bupivacaine	Cardiotoxicity, CNS toxicity	Antiarrhythmics (increase toxicity), calcium channel blockers (increase effects)	Cardiac arrhythmias, pregnancy	Monitor ECG in high-risk patients

Indications for antibiotic use in dental practice

Dental practitioners use antibiotics to treat acute odontogenic infections that affect dentoalveolar abscesses, periodontitis, and pericoronitis because these conditions can spread to other parts of the body, leading to cellulitis or osteomyelitis [23]. In such cases, penicillins (amoxicillin, amoxicillin-clavulanate) remain first-line agents, while clindamycin or azithromycin serve as alternatives in patients with penicillin allergies [24]. The severity of the infection, together with systemic involvement and microbiological factors, should guide the initial use of antibiotics to avoid unnecessary antibiotic consumption [25]. Preventive antibiotics use is recommended for patients at high risk who need invasive dental work, especially if they have prosthetic heart valves, past infective endocarditis, congenital heart defects, or immunocompromised conditions [26]. Both the American Heart Association and the American Dental Association endorse selective antibiotic prevention strategies to stop bacteremia-caused complications, including infective endocarditis [27]. Despite their therapeutic benefits, antibiotic stewardship is crucial to minimize the risks of antimicrobial resistance and adverse reactions, ensuring that prescriptions are evidence-based and reserved for cases where their use is essential [28].

Commonly used antibiotics and their spectrum

Dental infections require antibiotic treatment through various medications that exhibit diverse antibiotic ranges. Penicillin drugs, including amoxicillin and Augmentin, function as the preferred medicine for managing odontogenic infections because they effectively treat Gram-positive bacteria [29]. For individuals who have penicillin allergies, macrolides together with clindamycin act as suitable treatment options that handle both aerobic and anaerobic bacterial infections. Metronidazole and tetracyclines function as standard treatments for periodontal infections that occur in aggressive periodontitis and anaerobic environments [30]. The right choice of antibiotics remains essential as it protects against both bacterial resistance and unwanted side effects. Table 2 provides a summary of dental practice antibiotics, which includes their indications and spectrum, along with adverse effects and contraindications.

**Table 2:** Commonly Used Antibiotics and Their Spectrum GI: gastrointestinal; QT: QT interval (related to heart rhythm on an electrocardiogram); *C. difficile*: *Clostridioides difficile* (a bacterial infection causing severe diarrhea)

Antibiotic Class	Common Drugs	Spectrum of Activity	Indications in Dentistry	Adverse Effects	Contraindications
Penicillins	Amoxicillin	Gram-positive cocci, some Gram-negative	Odontogenic infections, dental abscesses	Hypersensitivity, GI distress	Penicillin allergy, renal impairment
	Augmentin (amoxicillin-clavulanate)	Broader than amoxicillin, includes beta-lactamase-producing bacteria	Recurrent infections, resistant bacterial strains	Diarrhea, hepatotoxicity	Liver disease, hypersensitivity
Macrolides	Erythromycin	Gram-positive aerobes, limited Gram-negative coverage	Alternative for penicillin-allergic patients	GI upset, QT prolongation	Liver dysfunction, history of QT prolongation
	Azithromycin	Broader than erythromycin, active against Gram-negative bacteria	Upper respiratory tract infections, prophylaxis	Fewer GI effects, prolonged QT	Severe hepatic impairment, pregnancy
Lincosamides	Clindamycin	Gram-positive anaerobes, some Gram-negative anaerobes	Severe odontogenic infections, bone infections	GI disturbances, *C. difficile* risk	History of colitis, liver dysfunction
Tetracyclines	Doxycycline	Broad-spectrum, including periodontal pathogens	Periodontitis, refractory cases	Photosensitivity, GI irritation	Pregnancy, children <8 years
	Minocycline	Effective against resistant periodontal pathogens	Aggressive periodontal infections	Vestibular toxicity, hepatotoxicity	Severe hepatic dysfunction, pregnancy
Nitroimidazoles	Metronidazole	Anaerobic bacteria, some protozoa	Anaerobic odontogenic infections, periodontitis	Metallic taste, nausea, neuropathy	Alcohol use, neurological disorders
	Tinidazole	Stronger activity against anaerobes than metronidazole	Severe anaerobic infections, periodontal therapy	Stronger neurotoxic effects, disulfiram reaction with alcohol	Alcohol use, severe liver disease

Alternative antimicrobial strategies

The dental community develops substitute antimicrobial methods that work together with typical antibiotics to combat antimicrobial resistance while enhancing dental health. Herbal medicine, alongside probiotics, demonstrates viable antimicrobial properties as part of its treatment approach. Medicinal plants such as green tea polyphenols, aloe vera, and neem extract exhibit antibacterial, anti-inflammatory, and biofilm-disrupting properties against oral pathogens [31]. The species of *Lactobacillus* and *Bifidobacterium* function as probiotics to reestablish microbial equilibrium and suppress pathogenic bacteria while strengthening the immune response, which proves beneficial for periodontitis and dental caries management [32].

The antimicrobial procedure called photodynamic therapy (PDT) employs photosensitizers that become active under light exposure to create reactive oxygen species, which successfully terminate oral bacteria, together with fungi and biofilms [33]. Medical research shows PDT delivers successful treatment outcomes in periodontal therapy, peri-implantitis management, and root canal disinfection because it exhibits high selectivity and low bacterial resistance [34]. PDT antimicrobial efficiency improves significantly when nanoparticles are integrated into the system because they enhance both photosensitizer penetration depth and agent stability [35]. Additional strategies for infection control need standardized clinical trials for achieving optimal effectiveness in dental practice [36].

Local anesthetics in dentistry

Local anesthesia stands as an essential dental technique that enables dental experts to perform various procedures such as extractions, periodontal surgeries, root canal treatments, and implant placements [37]. Nerve signaling transmission stops due to local anesthetics, which block sodium ion channels reversibly, thus providing powerful pain relief during dental procedures. The two primary local anesthetic groups include amides and esters, where in amides, lidocaine, articaine, bupivacaine, mepivacaine, and prilocaine are preferred because of their extended duration and minimal hypersensitivity risks [38]. The rapid metabolism of ester anesthetics by plasma esterases leads to increased allergic reactions, which restricts their use in modern dental practice.

Lidocaine remains the gold standard and primary choice for anesthesia since it starts working quickly and provides moderate time coverage while maintaining good safety benefits [39]. Also, the superior lipid solubility of articaine lets it spread efficiently into soft and hard tissues, thereby making it an ideal choice for infiltration anesthesia [40]. Bupivacaine represents the primary choice for extended procedures and pain control after surgery, yet doctors need to exercise caution since it poses elevated risks of heart toxicity [41]. Mepivacaine functions as an anesthetic with intermediate duration, along with weak vasodilatory effects that make it appropriate for patients who cannot use epinephrine [42]. Prilocaine stands out because of its low systemic toxicity profile, but medical providers select it mainly for patients at cardiovascular risk despite its link to methemoglobinemia at higher doses [43]. Local anesthetic selection in dental practices depends on onset speed, duration, and metabolism rate, together with individual patient needs to achieve maximum pain control without adverse reactions [44].

Factors affecting local anesthetic efficacy and safety considerations in dentistry

Local anesthetics in dentistry work through physiological, pharmacological, and technical factors to establish their ability to start working and persist in effect. The tissue pH stands as an essential factor because low pH levels observed in inflamed or infected tissue areas diminish the penetration of anesthesia while delaying its onset [45]. Local anesthetic effectiveness rises through vasoconstrictor administration as epinephrine lengthens duration and reduces systemic absorption and bleeding, while healthcare providers need to use caution when treating patients with cardiovascular diseases [41]. The success of local anesthetics depends on correct injection procedures that combine aspiration testing before administering the injection as well as proper needle positioning and targeted delivery adjacent to nerve structures [46].

The broad application of local anesthetics entails known potential side effects such as systemic toxicity, nerve injuries, and allergic reactions. The accumulation of high plasma concentrations in patients produces systemic toxicity that causes CNS depression and seizures along with cardiovascular collapse, especially when using bupivacaine and high-dose lidocaine [47]. Ester anesthetics produce more allergic reactions than amide anesthetics because of metabolites containing para-aminobenzoic acid (PABA) [48]. Patient complications such as persistent paresthesia and neuropathic pain can occur after intraneural injections and high anesthetic concentrations or from neurotoxic effects of articaine and certain other agents [49]. The delivery of local anesthesia requires healthcare providers to incorporate pH adjustment with vasoconstrictors alongside skilled injection methods and individual patient contraindications to reach maximum effectiveness and avoid adverse events.

Recent advances and innovations in local anesthesia

**Figure 3:**
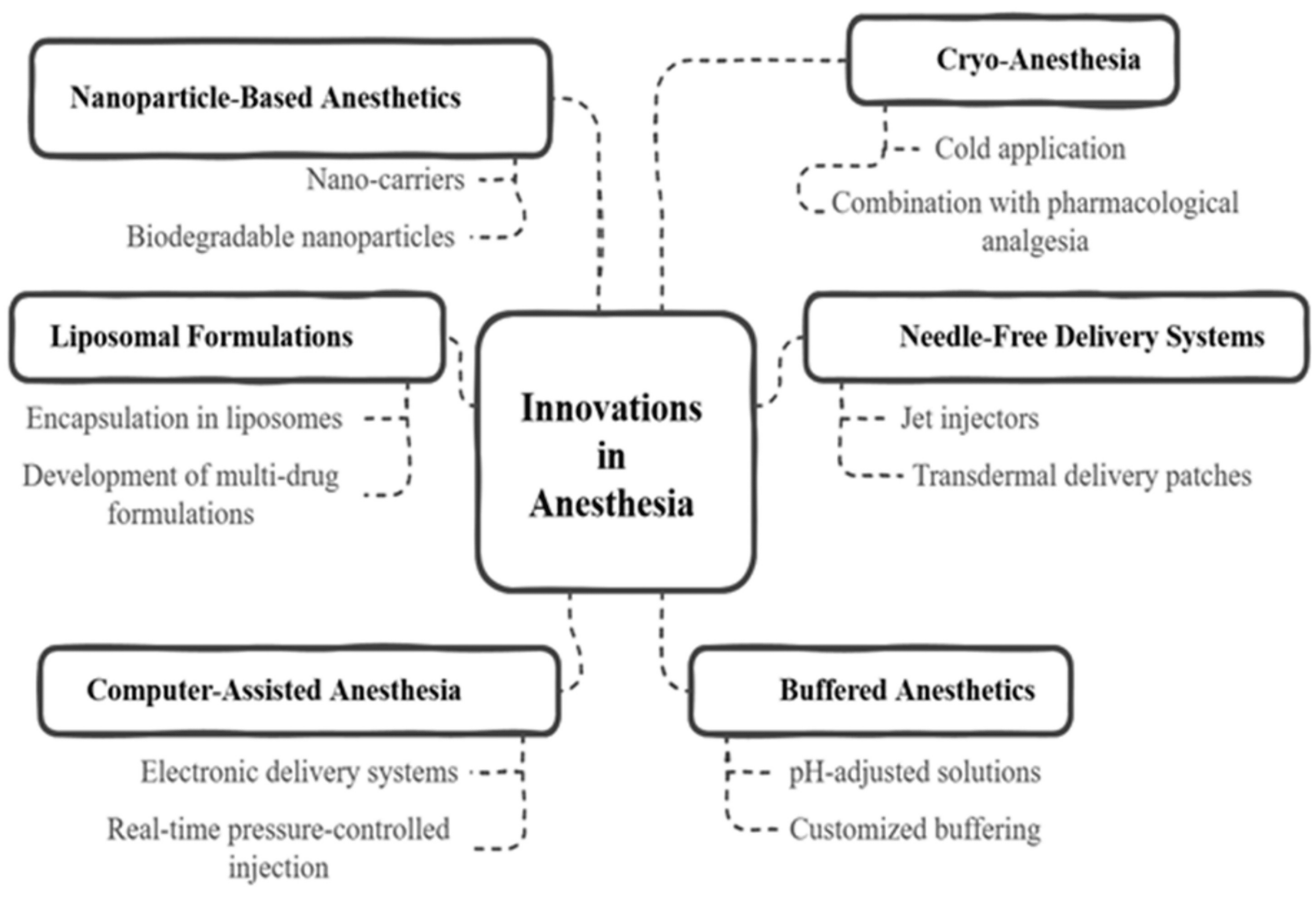
Recent Advances and Innovations in Local Anesthesia Credit: The image was created by the authors.

Local anesthesia innovations in dentistry practice have transformed pain control strategies by improving safety and effectiveness with increased patient-friendly procedures. Liposomal formulations deliver sustained-release drugs, which lead to longer anesthesia duration along with reduced toxicities in the body [50]. The use of jet injectors together with transdermal patches provides needle-free delivery systems that help patients who fear needles. The deployment of computer-assisted anesthesia provides exact drug delivery controlled by pressure, which reduces trauma to tissue [51]. The combination of buffered anesthetics with nanoparticle-based formulations helps drugs activate faster and enhances their availability to the body, and cryo-anesthesia gives pain relief without invasive procedures [52]. Figure 3 provides a summary of innovative techniques for local anesthesia alongside their benefits and restrictions, and their expected applications.

Future directions and research gaps in dental pharmacology

The development of pharmacology within dentistry depends on innovations related to personalized medicine, along with new drug formulations and better antimicrobial resistance strategies. Pharmacogenomics has established itself as a key technological approach in personalized dental pharmacology to create customized drug selections through genetic variation analysis of drug metabolism and its impact on both therapeutic effects and adverse side effects [53]. Genetic screening techniques improve both drug safety and effectiveness in analgesics, local anesthetics, and antibiotics, which reduces patient treatment failures and drug complications [54].

Scientific developments in drug formulation and delivery platforms work toward optimizing treatment results, reducing harmful effects on the whole body, and achieving better patient drug adherence [55]. Scientists have developed liposomal anesthetics together with nanoparticle-based antimicrobials and biodegradable drug carriers, which provide extended drug release and precise delivery while decreasing medicine applications and adverse effects [56]. Medical research has developed three new delivery platforms for dental medications, which include needle-free drug administration systems, intraoral controlled-release devices, and bioadhesive drug patches [57].

Antimicrobial resistance detection (AMR) in dental facilities continues to pose a critical worldwide healthcare problem. The improper use of antibiotics in dental practice alongside their excessive application has led to resistance growth among oral pathogens, thus requiring strong antibiotic stewardship policies. Phage therapy served as one example of the antimicrobial peptides and alternative antimicrobial agents that scientists are currently investigating to confront resistant bacterial strains while protecting the microbial ecosystem [58]. More research should focus on undertaking multidisciplinary research efforts by uniting genomic techniques with nanotechnology methods and bioinformatics systems to develop optimal dental pharmacotherapies with resistance reduction capabilities [24].

## Conclusions

This study highlights the critical role of pharmacology in modern dentistry, focusing on analgesics, antibiotics, and local anesthetics to ensure effective pain management, infection control, and procedural safety. NSAIDs and acetaminophen remain the first-line analgesics, whereas opioids are reserved for severe cases due to addiction risks. Antibiotics play a crucial role in treating odontogenic infections and preventing systemic complications, though antimicrobial resistance necessitates responsible prescribing practices. Local anesthetics, particularly lidocaine and articaine, have revolutionized pain-free dental procedures, with emerging innovations like liposomal formulations and computer-assisted anesthesia enhancing their efficacy. Despite advancements, challenges such as opioid dependency, antibiotic overuse, and variability in anesthetic response underscore the need for personalized pharmacotherapy. Future research should focus on pharmacogenomics, novel drug delivery systems, and antimicrobial stewardship programs to optimize dental pharmacology. By integrating evidence-based prescribing practices and emerging innovations, dentistry can achieve safer, more effective, and patient-specific pharmacological interventions, ultimately improving treatment outcomes and reducing complications.
